# P-2187. Emergency Department Patients’ Perceptions of the Seriousness of Hepatitis C – Implication on Improvement of HCV Care Continuum

**DOI:** 10.1093/ofid/ofae631.2341

**Published:** 2025-01-29

**Authors:** Dante Brown, Spencer Mann, Mun Heol Cho, Haley M Haines, Patrick Winguth, Lauren Cammer, Liz Peron, Wassim Bouhsane, Emma Hsieh, Kevin Feezel, Benjamen Riggan, Gaby Dashler, Richard E Rothman, Yu-Hsiang Hsieh

**Affiliations:** Johns Hopkins University School of Medicine, Bel Air, Maryland; Johns Hopkins University School of Medicine, Bel Air, Maryland; Johns Hopkins University School of Medicine, Bel Air, Maryland; Johns Hopkins University School of Medicine, Bel Air, Maryland; Johns Hopkins University School of Medicine, Bel Air, Maryland; Johns Hopkins University School of Medicine, Bel Air, Maryland; Johns Hopkins University School of Medicine, Bel Air, Maryland; Johns Hopkins University School of Medicine, Bel Air, Maryland; Johns Hopkins University School of Medicine, Bel Air, Maryland; Johns Hopkins University School of Medicine, Bel Air, Maryland; Johns Hopkins University School of Medicine, Bel Air, Maryland; Johns Hopkins School of Medicine, Baltimore, Maryland; John Hopkins University, Baltimore, Maryland; Johns Hopkins University, Baltimore, MD

## Abstract

**Background:**

U.S. urban emergency departments (EDs) have identified thousands of individuals infected with hepatitis C virus (HCV) through their testing programs. However, significant challenges remain with linkage to care (LTC) rates from EDs at < 30%, representing the biggest drop-off along the HCV care continuum (HCV CC). Leventhal’s Common-Sense Model of Illness Representations, a framework to understand people's health brief and responses to illness, could help narrow that gap by defining key factors that contribute to self-perceived view of hepatitis C seriousness, and develop tools to improve the HCV CC.

**Methods:**

Secondary data analysis of an ongoing randomized controlled trial of an individualized HCV educational web-based tool was performed. Patients presenting to an urban ED in Baltimore, Maryland from September 2022 to March 2024, ≥ 18 years, with a documented positive HCV Ab and no history of treatment (excluding those treated prior to reinfection) were eligible. The tool collected sociodemographic characteristics, historical healthcare utilization, HCV knowledge level, perceived barriers to care, and motivation level to care. Perceived seriousness of HCV was measured via an eight 5-point Likert scale which assessed perceptions of control, concern, awareness, and impact of HCV. Multivariable linear regression analysis was performed to determine factors associated with perceived seriousness of HCV.

**Results:**

79 (87%) of 91 participants completed the baseline survey and were included for analysis. The majority were ≥40 years (71%), male (72%), or self-identified black (62%). Mean perceived seriousness of HCV was 3.2±0.7. A higher score was positively associated with the Black race, perceived barrier to HCV care score, and perceived motivation level for HCV care score [regression coefficient: 0.38±0.13; 0.30±0.10; 0.33±0.11, respectively; all p< .05] after adjusting for confounders.

**Conclusion:**

We observed that amongst HCV-infected ED patients who were not currently engaged in care, there was an overall neutral perception of their HCV illness. Factors associated with higher score in perceived seriousness reflect current HCV and opioid intersecting epidemic and suggest there are mutable health systems issues that can be addressed.

**Disclosures:**

Richard E. Rothman, MD, PhD, abbvie: Grant/Research Support|CEPHEID: Advisor/Consultant|CEPHEID: Grant/Research Support|CEPHEID: Honoraria Yu-Hsiang Hsieh, PhD, AbbVie: Grant/Research Support

